# Study of the Use of Permethrin 5% Cream in Galicia (Spain) between 2018 and 2021

**DOI:** 10.3390/idr15020023

**Published:** 2023-04-19

**Authors:** Severo Vázquez-Prieto, Antonio Vaamonde, Esperanza Paniagua

**Affiliations:** 1 Universidad de Los Lagos, Osorno, Chile; 2 Vicerrectoría de Investigación y Postgrado, Universidad Católica del Maule, Talca, Chile; 3 Departamento de Estadística e Investigación Operativa, Universidad de Vigo, 36310 Vigo, Spain; 4 Laboratorio de Parasitología, Departamento de Microbiología y Parasitología, Facultad de Farmacia, Universidad de Santiago de Compostela, Campus Vida, 15782 Santiago de Compostela, Spain; 5 Instituto de Investigación en Análisis Químicos y Biológicos (IAQBUS), Universidad de Santiago de Compostela, 15782 Santiago de Compostela, Spain

**Keywords:** drug utilization study, scabies, defined daily dose, pyrethroid

## Abstract

Drug utilization studies can provide direct insights into how a drug is used in real-world conditions and can give a rough estimate of the proportion of the study population treated with it. In the present work, we examined the consumption of permethrin 5% cream in the four provinces of Galicia (a Spanish autonomous community) and described the seasonal variability and the annual evolution of its consumption between 2018 and 2021. A descriptive, cross-sectional, and retrospective study of the consumption of this drug, expressed in defined daily dose per 1000 inhabitants per day (DID), was carried out. The results obtained revealed differences between the amounts consumed in the four Galician provinces (*p* < 0.001). No specific geographical pattern was observed; however, the results suggested a marked seasonality and a slightly increasing global trend in the consumption of permethrin 5% cream throughout the study period. Since the only authorized indication of this drug in the study area is the treatment of scabies, this work may give an idea of the epidemiological situation of the disease in Galicia and serve to establish public health strategies against this parasitosis.

## 1. Introduction

Human scabies is an ectoparasitosis caused by the mite *Sarcoptes scabiei* var. *hominis* (Acari: Sarcoptidae). It is transmitted by prolonged direct contact (at least 5 to 10 min) with an infected person or less frequently through contaminated fomites when hosts have a high parasite load, such as Norwegian scabies (an extreme form of the disease, seen mainly in the elderly and immunosuppressed, that is manifested by severe hyperkeratosis with frequent fissures and secondary infections) [1,2]. The scabies mite is an obligate parasite that penetrates, resides, and reproduces on human skin. The fertilized female mite burrows into the infected individual and lives for 10 to 14 days in the epidermis of the skin, laying three to five eggs per day in the stratum corneum. Within three days, the eggs hatch into larvae, then develop into protonymphs, and two to three days later, they develop into tritonymphs. After four to seven days, adult mites are present. There is an incubation period of four to six weeks, in which the new host may be asymptomatic and unaware that they are infested, during which time they can still transmit scabies to new hosts. Subsequent infestations produce symptoms within days, as the host will have developed hypersensitivity to the parasite [3]. The main symptoms of scabies include intense itching, which is usually worse at night, bumpy, pimple-like rashes with small blisters or scales, and small curved and raised lines on the skin. The most frequent locations are the flexor surfaces of the elbows, armpits, hands, interdigital grooves, wrist, groin, dorsal surface of the foot, genitals, buttocks, and gluteal and inguinal folds. The head, face, neck, palms of the hands, and soles of the feet are often affected in infants and very young children, but usually not in adults or older children [4,5,6,7].

Epidemiologically, the global prevalence of scabies is estimated to be 200 to 300 million people, with wide variations among specific geographic regions. In 2017, scabies was added to the World Health Organization list of Neglected Tropical Disease. It causes significant socioeconomic impacts and affects all ages, sexes and types of social class and educational levels, although the risk of transmission increases in areas where hygienic conditions are not optimal and in settings with high concentrations of people where skin-to skin contact is common, such as kindergartens, schools, hospitals, nursing homes, and other closed institutions (prisons, military and refugee camps, mental institutions, etc.) [7,8,9]. The diagnosis of a scabies infestation is usually made from the history and examination of the affected person. It is important to make a correct differential diagnosis with common skin problems that have symptoms similar to scabies (e.g., eczema, prurigo, drug-related eruptions, and psoriasis), which are usually treated with topical corticosteroids, since the administration of topical corticosteroids significantly worsens the state of the disease [10]. A definite diagnosis is established by examining the mites, eggs, or feces under a microscope after scraping the lesions with a scalpel blade and mixing them with a mineral oil solution (detection rates vary from 10 to 70%) [11]. Dermatoscopes have eliminated the need for invasive skin scrapping; however, they are not affordable in all regions, cannot visualize feces or eggs, and have more difficulty detecting mites in darker skin types [3].

Regarding treatment, permethrin 5% cream is recommended as the first option in various international guidelines [1,12,13]. The cream is applied topically to the patient’s dry skin from head to toe and under the fingernails, is washed off after 8 to 14 h, and is often reapplied in the same way one week later. Oral ivermectin (six mg, two doses: on day 1 and 15 days later) can be used as an alternative treatment. The simultaneous treatment of all the contacts of the infected patient is recommended, whether or not they have symptoms, as well as washing at high temperatures (at least 60 °C), or storing underwear, clothing, and bedding that have been in contact with the patient in the 72 h prior to treatment in closed plastic bags for three to eight days [6,11].

A national epidemiological study on scabies has recently been carried out in Spain. The authors used four databases that record information from various perspectives: hospital admissions, patients who attended a primary healthcare service, outbreaks, and occupational diseases. According to primary care databases, children and young adults were the most affected, whereas the elderly made up the majority of patients admitted to hospitals and cases reported in outbreaks. Most of the outbreaks occurred in homes and nursing homes, but military barracks, healthcare settings, and nursing homes had the highest number of cases per outbreak. Healthcare workers were the most frequently affected professional group, with the majority of occupational cases also occurring in social services and healthcare settings. Scabies admissions trended downward from 1997 to 2014 (annual percentage change, APC = −11.2%) and then increased from 2014 to 2017 (APC = 23.6%) [14].

Galicia is a Spanish autonomous community located in the northwest of the Iberian Peninsula. It has a total area of 29,574 km^2^ and is made up of four provinces: A Coruña, Lugo, Ourense, and Pontevedra. According to the study carried out by Redondo-Bravo et al. [14], Galicia was the third autonomous community with the highest incidence of scabies during the period from 2011 to 2017, according to an analysis of the CMBD (data from hospital admissions discharges) and RENAVE (outbreaks data from the Spanish Surveillance Network) databases, and the second of those according to an analysis of the ODR (occupational diseases recorded and followed up by the National Social Security System) database. In the present work, we approached the study of this disease in Galicia by analyzing the consumption of permethrin 5% cream using the methodology of drug utilization studies. This type of study can provide direct knowledge of how a drug is used in real practice and, through the use of certain quantitative parameters, can give an approximate idea of the volume of the population treated daily with the usual dose of a given drug, as well as allowing comparisons of consumption between different geographical areas or within the same area across different periods of time, regardless of any differences or changes in price or presentations [15,16,17].

The objectives of this study were: (a) to examine the consumption of permethrin 5% cream in the four provinces of Galicia between 2018 and 2021, and (b) to describe the seasonal variability and the annual evolution of its consumption during this period.

## 2. Materials and Methods

We carried out a descriptive, cross-sectional, and retrospective study of the consumption of 5% permethrin cream (group P03AC04 of the Anatomical Therapeutic Chemical classification system) in the four Galician provinces between 1 January 2018 and 31 December 2021. The only authorized indication of this drug in the study area is the treatment of scabies. Consumption information was obtained through the General Subdirectorate of Pharmacy of the Galician Health Service (SERGAS) from the monthly billing database of official medical prescriptions dispensed in the Galician pharmacy offices.

The methodology used was similar to that described in our previous study [16]. The measure of consumption was the defined daily dose (DDD) expressed as the number of DDDs per 1000 inhabitants per day (DID):

$$\mathrm{DID}=\frac{n^{⍛}\mathrm{DDD}\times 1000}{\mathrm{Population}\times t}$$

where *t* corresponds to the number of days in the month and population is the number of inhabitants in each province associated with the year the drug was dispensed. The annual population figures were obtained from the Galician Institute of Statistics [18].

For information processing, a dataset was created in the Excel program. The statistical analysis was performed using the free-use statistical program R version 4.2.1. The means and 95% confidence intervals (95% CI) were analyzed. The comparison of consumption between provinces was carried out using the non-parametric Kruskal–Wallis test. In order to calculate the seasonal index, a classical time series decomposition by moving averages was used (seasonal, trend, and irregular components) with a multiplicative model. In general, a significance level of 0.05 was used.

## 3. Results and Discussion

Scabies is a significant and common health problem. It is a highly contagious parasitic skin disease that negatively affects quality of life and can cause psychosocial problems for patients and their families. Due to the increasing trend of its prevalence and the lack of its clinical recognition, the WHO encourages countries to stop this trend by 2030 as part of its Sustainable Development Goals [9]. Here, we conducted one of the first studies focused on investigating the consumption of permethrin 5% cream in the world. In the literature review, we only found one recent report on the sales of four scabicidal agents, including permethrin 5% cream, before and during the COVID-19 pandemic in Israel. As a limitation, the results of this study were based on the sales of one pharmacy chain in the country [19].

**Figure 1:**
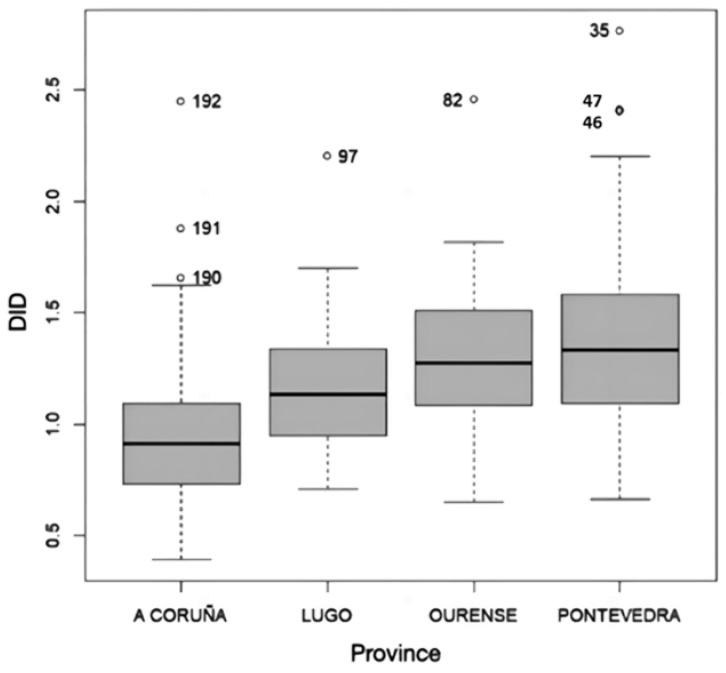
Distribution of defined daily dose per 1000 inhabitants per day (DID) by province.

The distribution of DID by provinces can be seen in Figure 1. The results obtained revealed differences between the mean values of DID in the four Galician provinces (*p* < 0.001), with Pontevedra and A Coruña being the ones with the highest and lowest consumption, respectively (Table 1). However, no specific geographical pattern was observed.

The differences found in the consumption of permethrin 5% cream may be explained by demographic patterns, such as the presence of institutionalized people, immigrants living in poor conditions, or people at risk of social exclusion [14]. It is important to note that the occurrence of this parasitosis is highly influenced by the living conditions of the dwellings (especially overcrowding) and other socioeconomic factors, including poverty or a lack of access to healthcare [2]. Thus, several authors have demonstrated a significant increase in incidence during the lockdown period due to SARS-CoV-2 compared to the previous stage. In Italy, 12.1% of cases were reported between 22 February and 3 May 2020, compared to 3% in the same period in 2019 [20]. In Spain, 64 cases were reported during the period of home confinement (March–May 2020), compared to an average of 18.6 cases during the same period in the five years prior to the pandemic. In addition, more than 80% of patients with scabies had a history of family members or cohabitants who were diagnosed with scabies or highly suspected of having it. Furthermore, more than half of these patients consulted the dermatologist accompanied by their cohabitants, thus forming “family clusters” [21]. In a prospective study in pediatric patients who attended a dermatological clinic for a general dermatological examination, Herzum et al. found that 83% of the patients diagnosed with scabies lived in popular districts, with multiple family components (4.5) living all under the same household. Furthermore, 66% of these patients were part of blended families [22]. On the other hand, the demographic differences in the population pyramid at the provincial level could also have influenced the geographical differences observed [14,18]; however, the collated prescription data did not provide details on the age or gender characteristics of the patients.

**Figure 2:**
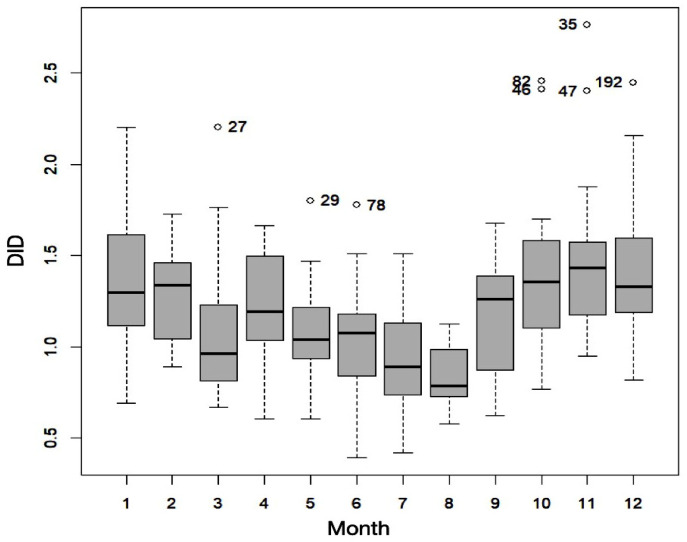
Monthly evolution of the defined daily dose per 1000 inhabitants per day (DID) during the period studied (2018–2021).

The results obtained suggested marked seasonality (*p* < 0.001), with higher consumption of permethrin 5% cream in the autumn and winter months. In Figure 2, we can observe the monthly evolution during the period studied, with lower values in summer and higher values in winter. In terms of the seasonal indices, the month with the highest seasonality was November, which exceeded the annual average by 39.8%, and the month with the lowest seasonality was July (41.1% below the average). The entire May–October semester had low seasonality (October practically coincides with the average), and the November–April semester had high seasonality (see Table S1 in Supplementary Materials). These results are consistent with previous studies linking high incidences of scabies infestations with cold temperatures and humidity. An epidemiological study of the seasonal trends of scabies in the Israeli Defense Forces showed a higher incidence of infestation during the cooler months of the year [23]. In Taiwan, the incidence of scabies was negatively correlated with temperature (*p* < 0.001) and positively correlated with humidity (*p* < 0.001) [24]. These seasonal variations may also be associated with alterations in human behaviors. For example, the higher number of rainy days in autumn and winter seasons may cause people to stay indoors, leading to overcrowding and higher levels of contact with other people. This increased personal contact may, in turn, increase the risk of transmission of the parasite [24]. Finally, the fertility and survival of mites can also be increased in cold weather [25].

**Figure 3:**
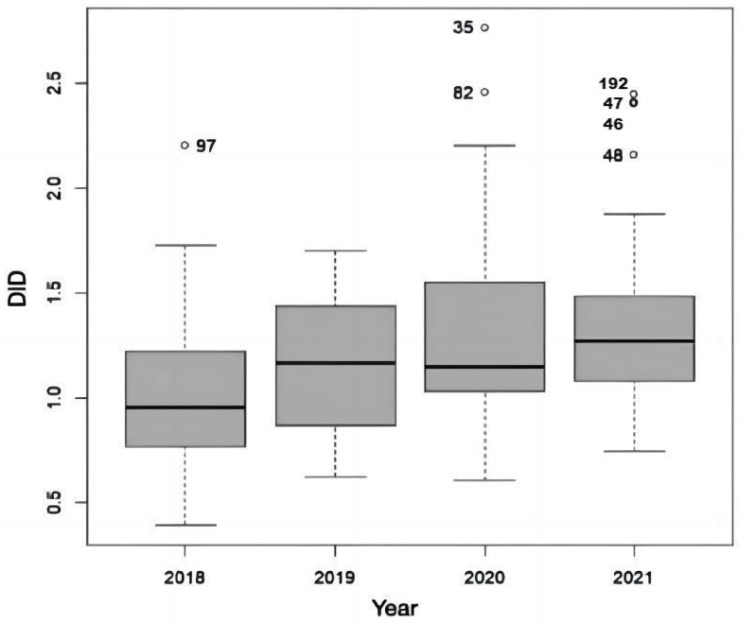
Annual evolution of the defined daily dose per 1000 inhabitants per day (DID) from 2018 to 2021 in Galicia (Spain).

A slightly increasing trend in the consumption of permethrin 5% cream in Galicia throughout the study period can be seen in Figure 3. The Kruskal–Wallis test allowed us to establish that, on average, the variable was different in different years (*p* < 0.001). The first year was significantly different from the last one (see Table S2 in Supplementary Materials). This result agrees with those from a study conducted in Israel that reported a constant and significant increasing trend in annual scabicide sales from 7767 units in 2010 to 21,640 in 2021, averaging 1261 units per year, whereas the average annual increase was 0.12 units per 1000 people, increasing from 1.01 units/1000 people in 2010 to 2.29 units/1000 people in 2021 [19]. There are several possible explanations for our findings. The main reason could be treatment failure, which has generally been attributed to various causes, such as incorrect application of the cream by patients, failure to simultaneously treat cohabitants, or insufficient disinfection of the environment [26,27,28]. However, an increasing number of dermatologists are suggesting a real resistance to permethrin after ruling out the previously described errors and achieving a cure with other active principles for topical use, including magistral preparations, such as sulfur in an ointment base (petrolatum) or benzyl benzoate [29]. For example, Sunderkötter et al. warned in 2018 of a surprising increase in the incidence of scabies in Germany and described, among the possible causes, the development of resistance to permethrin [30]. In a randomized clinical trial conducted in Austria, a cure rate of only 29% was observed after two applications of permethrin 5% cream one week apart [31]. In Italy, 21 of the 31 symptomatic patients did not respond to the usual regimen of this drug and needed other therapies [32]. In another study in Italy, 96 of 155 patients did not achieve remission of parasitosis, despite using a more intensive regimen of permethrin application [33]. Recently, Bassi et al. reported a 65% resistance rate to permethrin 5% cream after surveying 317 Italian dermatologists [34]. It is interesting to note that the resistance of mites to permethrin was demonstrated years ago in in vitro studies. The results obtained by Walton et al. indicated that 35% of mites were still alive after 3 h, and 4% after 18 to 22 h of constant exposure to the drug [35]. Among the different mechanisms that have been postulated as responsible for the resistance of *S. scabiei* to permethrin, there is an increase in the activity or expression of the enzyme glutathione-S-transferase, an increase in the expression of ATP-binding cassette transporters, such as the multidrug-resistant protein, and mutations in voltage-gated sodium channels or ligand-gated chloride channels [36].

The increasing trend observed in the consumption of permethrin 5% cream in Galicia throughout the study period could also be related, to a lesser extent, to delayed diagnosis due to the saturation of primary care, resulting in a greater parasitic load and capacity for infection of the host, as well as a longer contagious period. On the other hand, cuts in social and health services, in addition to the worsening of living conditions as a result of the economic deterioration of certain layers of society, may also be playing some kind of role [14]. Other factors could also contribute to this phenomenon, such as increases in the immunocompromised population, the elderly requiring assistance, immigration, and refugees [26,30].

There are some limitations to this study. First, although the DDD is a drug consumption unit with many advantages over others, it has some limitations that must be taken into account when interpreting the results of a study in which that unit is used. It does not necessarily correspond to the prescribed daily dose and/or the amount of medicine consumed by the patient in practice [17]. Second, the number of patients dispensing without a medical prescription or with a private prescription is unknown. However, it is estimated that this percentage should be low due to the obligation to present a medical prescription for dispensing in pharmacies. On the other hand, the prescription of this drug through private entities is marginal (Pharmacy Carmen Goicoa Gago, personal communication), so it can be estimated that the presented data provide a close approximation to reality [16].

In conclusion, the present study showed differences between the amounts of permethrin 5% cream consumed in the four Galician provinces, as well as marked seasonal variation and a slightly increased global trend in its consumption between 2018 and 2021, which can be explained by multiple factors. The results obtained in this study cannot be generalized to other geographic contexts, since many factors (e.g., living accommodation and subsequent interpersonal contact, age distribution, or general health) may differ substantially between them; however, they may give an idea of the epidemiological situation of scabies in Galicia and serve to establish public health strategies against this parasitosis. Thus, in line with what was expressed by Redondo-Bravo et al. [14], measures, such as accurate data collection, proper disease reporting, improved clinical diagnosis, early detection, proper and timely case management, improved hygiene, staff training, and the wide implementation of scabies treatment (considering mass drug administration in institutional outbreaks), should be taken into account in order to mitigate the impact of scabies in the Galician population. In addition, reducing humidity and increasing room temperatures could also help to reduce scabies infestation rates [24]. Finally, adequate education and accessible healthcare may be the pillars of outbreak prevention [9]. More studies are needed to clarify the determining factors of the infection and those that can explain the observed results. This will require a multidisciplinary approach with contributions from different areas of knowledge, such as epidemiology, sociology, and public health.

## Figures and Tables

**Table 1:** Mean values and confidence intervals (95% CI) of the defined daily dose per 1000 inhabitants per day (DID) by province.

	Mean DID	95% CI
A CORUÑA	0.967	0.860, 1.074
LUGO	1.168	1.087, 1.250
OURENSE	1.279	1.177, 1.381
PONTEVEDRA	1.377	1.245, 1.510

## Data Availability

Additional data is available from the corresponding authors on reasonable request.

## Supplementary Materials

The following supporting information can be downloaded at: https://www.mdpi.com/article/10.3390/idr15020023/s1, Table S1: Seasonal index (SI) by month in Galicia from 2018 to 2021 (the values are expressed in relation to the unit, which represents the annual average). Table S2: Mean values and standard deviation (SD) of the defined daily dose per 1000 inhabitants per day (DID) by year (2018–2021) in Galicia.
