# Supplementary material for: Study of the Use of Permethrin 5% Cream in Galicia (Spain) between 2018 and 2021
Source: Infect Dis Rep. 2023 Apr 19;15(2):222–30. doi: 10.3390/idr15020023 (PMC10138315; doi:10.3390/idr15020023)
Supplement: Supplementary file 1 [file idr-15-00023-s001.zip › idr-2095256-supplementary.pdf]

Tabla S1. Seasonal index (SI) by month in Galicia from 2018 to 2021 (the values are expressed in relation to the unit, which represents the annual average).

Month	SI
January	1.188
February	1.069
March	1.133
April	1.072
May	0.965
June	0.82
July	0.589
August	0.72
September	0.93
October	0.992
November	1.398
December	1.122

Table S2. Mean values and standard deviation (SD) of the defined daily dose per 1000 inhabitants and day (DID) by year (2018-2021) in Galicia.

Year	Mean DID	SD DID
2018	1.016	1.016
2019	1.165	1.165
2020	1.273	1.273
2021	1.338	1.338
